# Maternal intravenous fluids and postpartum breast changes: a pilot observational study

**DOI:** 10.1186/s13006-015-0043-8

**Published:** 2015-06-02

**Authors:** Sonya Kujawa-Myles, Joy Noel-Weiss, Sandra Dunn, Wendy E Peterson, Kermaline Jean Cotterman

**Affiliations:** School of Nursing, Faculty of Health Sciences, University of Ottawa, Ottawa, Ontario Canada; Better Outcomes Registry & Network (BORN Ontario), Ottawa, Ontario Canada; Children’s Hospital of Eastern Ontario Research Institute, Ottawa, Ontario Canada

**Keywords:** Breastfeeding, Engorgement, Edema, Lactogenesis II, Intravenous fluids

## Abstract

**Background:**

The current breastfeeding initiation rate in Canada is approximately 87%. By one month, about 21% of women have stopped breastfeeding. Engorgement and edema in breast tissue can lead to breastfeeding challenges which may contribute to early weaning. The aims of this pilot research study were to explore the relationship between intrapartum intravenous fluids given to mothers and postpartum breast swelling in the first 10 days postpartum and to determine if a larger study was warranted and feasible.

**Methods:**

A prospective, longitudinal, observational cohort pilot study with repeated measures and a within-subjects design was completed. Participants were first time mothers who have a single, healthy newborn and had a spontaneous vaginal birth. Daily data collection from admission into the study until postpartum day 10 took place. Descriptive statistics are reported and linear regression analysis was used to model the relationship between IV therapy and postpartum breast edema.

**Results:**

Women who received intravenous fluids during labour had higher levels of breast edema postpartum and rated their breasts as firmer and more tender than women who did not receive intravenous fluids. Participants who had intravenous fluids described patterns of fullness that appeared to be related to edema as opposed to fullness associated with engorgement and lactogenesis II.

**Conclusions:**

The findings demonstrate that mothers in this pilot study who received intravenous fluids in labour and postpartum had higher levels of breast edema. These results suggest a larger study is warranted to more fully examine the effects of intravenous fluids on postpartum breast swelling.

**Electronic supplementary material:**

The online version of this article (doi:10.1186/s13006-015-0043-8) contains supplementary material, which is available to authorized users.

## Background

Breastfeeding, a normal physiological behaviour, is not without challenges which can lead to high rates of weaning. Breastfeeding initiation rates in Canada are currently about 87.3% but 21.4% of women stop breastfeeding by one month [1,2]. Key reasons given for early weaning are sore breasts and painful nipples [3,4]. Postpartum breast engorgement and breast edema are two forms of postpartum breast swelling which can contribute to sore breasts and nipple trauma [3,4]. Engorgement is defined as overfull breasts due to excess milk and increased blood supply, whereas edema is the result of increased fluid in the interstitial space [5,6].

Postpartum breast swelling may contribute to breast and nipple pain, nipple damage, breast infections, and may be one of the reasons women stop breastfeeding [3,4]. Breast swelling may cause difficulties for babies trying to latch due to swollen breast tissue not being supple enough to achieve a deep latch and feed without causing nipple damage [7,8], may reduce the amount of milk a baby transfers at breast [9], and may decrease long term milk supply [3].

While there have been some studies about postpartum engorgement, postpartum breast edema is not well defined or studied. If the etiology and management of postpartum breast swelling was better understood, clinicians and mothers may be better able to minimize postpartum breast swelling and its consequences, potentially improving breastfeeding rates in the first few weeks postpartum and increasing the likelihood mothers would achieve their breastfeeding goals.

The purpose of this pilot study was to explore the relationship between intravenous (IV) fluids given to mothers during the peripartum period and postpartum breast swelling. This pilot study was also conducted to determine if a larger study is warranted and if so, which data collection protocols would be most useful in a larger study. The research question developed for the study was, “What is the relationship between the amount of IV fluids given to primiparous women and the edema of the breast and areola complex experienced by breastfeeding women in the first 10 days postpartum?”

A narrative literature review [10] was completed to learn what is known about postpartum breast engorgement and breast edema and to determine how these phenomena are defined. The literature is unclear when it comes to distinguishing between postpartum breast engorgement and breast edema. Postpartum breast edema may be a new or previously unidentified phenomenon related to medicalized birth practices. It was uncommon to see edema in postpartum mothers when studies of engorgement first appeared [5].

The two conditions are not identified as being different in the literature, but rather they are described as being two parts of the same phenomenon [3,4]. The effects of IV fluids given to mothers during labour have not been extensively studied, and there is limited research on the effects of IV fluids on postpartum breast engorgement or breast edema. Studies on postpartum engorgement failed to take IV fluids administered to mothers into account [9,11,12] and the one study found on postpartum edema failed to look at breast edema, but rather focused on peripheral edema [13].

Factors associated with engorgement include: delayed initiation of breastfeeding, infrequent breastfeeding, limiting duration of breastfeeds, late maturation of milk, and supplementary feeds given to baby [11]. Factors that contribute to peripheral edema seem to be different from but related to the factors that contribute to engorgement. Mothers who experience pregnancy induced hypertension, oxytocin induced labours, and a large amount of IV fluid during labour are at an increased risk for peripheral edema in the postpartum period [14]. Mothers who receive IV fluids have been shown to have decreased blood osmotic pressure causing peripheral edema [13,15]. A study about infant weight loss found a significant positive correlation between total IV fluids given to a mother during labour and a delay in lactogenesis II [16]. Mothers who receive IV fluids may be at an increased risk for postpartum breast edema, and may experience a delay in lactogenesis II [13,16].

This pilot study explored postpartum breast swelling. It is a phenomena with little known cause and outcomes. Increased knowledge about postpartum breast swelling should help clinicians and parents understand its impact on the postpartum experience, including breast comfort, milk production, and breastfeeding experience. Appropriate interventions can then be developed to help minimize the negative impact of this swelling.

## Methods

This study was a prospective, longitudinal, exploratory, observational cohort pilot study with a convenience sample, repeated measures, and a within-subjects design. Due to the limited amount of information found during the literature review, a pilot study was conducted to explore this topic further. Ethics approval for this study and all amendments were granted by the University of Ottawa Office of Research Ethics and Integrity (file number H08-12-09) and from the hospitals where the study took place. The two hospitals are both level 2 community hospitals, and are run by the same organization. There are approximately 3000 births per year between the two sites (900 at one site and 2100 at the other site). SPSS 21 statistical software was used for descriptive statistics and tests of significance. Descriptive statistics were used to summarize demographic characteristics of the study population. For this study, day of birth was considered Day 0, and babies entered into Day 1 24 hours after birth.

Eligible participants were primiparous women who gave birth vaginally following spontaneous labour to a single, full term, healthy infant, and who planned to exclusively breastfeed. Primiparity was a requirement due to the effects of multiparity on postpartum breast changes [9]. Healthy was defined as mother and baby who were discharged home together with no contraindications to exclusive, unrestricted breastfeeding. Participants had to be able to read, write, and speak English and had to live in the same geographical area as the primary researcher (S.K-M.) to allow for home visits.

Exclusion criteria were any factors that may have affected exclusive breastfeeding (e.g., mother baby separation, newborn facial anomalies, no breast growth during pregnancy, medically induced labour). Data were collected daily from recruitment before birth (to determine baseline measurements) until ten days postpartum. Ten days was chosen as the timeframe for this study, as 90% of women will experience engorgement and its resolution during this period [9].

Data collection included information about possible moderators and mediators. Moderators which might have an effect on swelling include early breastfeeding, frequent breastfeeding, previous breastfeeding experience, maternal self-efficacy and the overall duration of feeds [9,10]. Mediators which might contribute to swelling include parity, type of birth, medication given during labour and birthing, choice of anaesthesia, gestational age, and timing and type of IV fluids [12,13].

Steps were taken to minimize bias. For this pilot study subject bias could not be avoided due to the small sample, therefore the sample might not represent the population and be generalizable [17]. Measurement bias was avoided by having the same researcher perform all measurements using clinically approved tools. The baby scale was checked by using a standardized weight prior to every weight measurement to ensure reliability.

Sample size was determined by referring to other studies on engorgement. The majority of sample sizes were between 6 and 54, with 6 studies having 20 or less participants. It was deemed reasonable that a final sample size of 20 for this pilot study would be sufficient to achieve the study goals.

Participants were asked to complete prenatal and postnatal questionnaires to collect basic demographic data. Data regarding IV fluids were collected prospectively during the intrapartum period by nurses at shift change or more frequently if possible. Volume and type of IV fluids were tracked from commencement of IV therapy until it was discontinued. These data were collected using milliliters.

A classic edema rating scale measuring 1+ to 4+ was used to measure breast and areola edema [18]. Edema was quantified by determining the ‘depth of impression’ and ‘time to rebound’. Depth of impression was measured by applying gentle pressure with an index finger pad on areola tissue just above the nipple and again about two inches above the nipple on a mother’s breast for 5 seconds. Depth of impression and time to rebound was noted visually and level of edema rated. This scale has been found to have poor inter-rater reliability [19], however, the primary researcher (S.K-M.) collected all data for this variable ensuring internal consistency. Peripheral edema was measured on the last 2 participants by taking wrist and ankle measurements with a tape measure. This measurement was taken to track overall edema levels and to monitor resolution of peripheral edema with the idea of comparing it with resolution of breast edema.

Initially edema was assessed by sight and not confirmed by touch. For the first four participants, all of which had IV fluids, S.K-M. measured edema by observing for visible rebound of skin tissue. However, participants had excellent skin turgor and skin would rebound faster than the underlying tissue. To identify edema, the skin on a women's breast or areola needed to be palpated to determine if the underlying tissue had rebounded. Beginning with Participant 5, the researcher gently pressed for 5 seconds, and then she ran her finger over the site to determine if an indent remained below the visible skin. After the initial press and release, she checked every 5 seconds to see if the area of indentation underneath had rebounded. Palpation, rather than visualization, provided a better assessment of edema by determining what was happening with the underlying tissue. As a result, the edema ratings with the first four participants were lower than they might have been with palpation.

For statistical analysis the two breast and areola scores were averaged into one daily score for each participant resulting in repeated measures over the 11 data collection points. A linear regression was run using IV therapy (independent variable, yes/no) against these daily edema scores (dependent variable) for the 17 participants.

Participants were asked to rate the degree of breast firmness and tenderness at the time of data collection according to a 6 point scale adapted by Hill and Humenick for their research on breast engorgement [9] See Table 1.

**Table 1:** **Maternal breast self-assessment scale**

1. Soft, no change	4. Firm, beginning tenderness
2. Slight change	5. Firm, tender
3. Firm, non-tender	6. Very firm and very tender

To determine if onset of lactogenesis II was occurring, mothers were also asked, “Do you feel your milk has come in - signs of this may include breasts feeling fuller, heavier, tender, and leaking milk?” [20]. A mother’s perception of the onset of lactogenesis II has been found to be accurate [21].

Newborn weights were measured on a daily basis, performed by mothers and supervised by S.K-M. Other measurements taken included milk maturation using the Milk Maturation Index of Colostrum and Milk (MICAM) [22], information on how baby was latching, nipple and areola height and width (measured in millimeters), and pumping and supplementing (by asking participants to recall their actions over the last 24 hours).

## Results

Recruitment took place from November 2012 to November 2013. Twenty-five women were recruited, eight participants did not continue to meet the inclusion criteria following birth leaving 17 mothers remaining in the study. One participant was lost to follow up, but all available data collected was included in the analysis. The attrition rate of 33% was higher than the expected 25% and mostly due to mother baby separation (n = 5) and participants having a Caesarean section (n = 3). The majority of women (12; 71%) had IV therapy, an epidural, and oxytocin. The policy at both hospitals is to give a bolus of 500mls of IV Normal Saline prior to insertion of epidural analgesia and then to have a continuous infusion of Normal Saline running at 125mls per hour until after birth. IV fluids can be discontinued after a vaginal birth once the mothers’ fundus is firm and vaginal bleeding is within normal limits. One participant had only IV therapy, and 4 participants had no interventions during their labour and birth. In total 13 of the 17 (76%) participants had IV fluids in labour and post birth. Table 2 shows the characteristics of participating mothers and newborns.

**Table 2:** **Characteristics of participating mothers and newborns (n = 17)**

**Characteristics**	**Mean (range)**	**Frequency (%)**
Maternal age (years)	30.1 (20–37)	
In committed relationship*		16 (94)
Completed post-secondary education		15 (88)
Family income >78 K (CAN)		11 (65)
First language		
English		14 (82)
French		0 (0)
Other		3 (18)
Noted prenatal breast growth		
Yes		14 (82)
Previous breast surgery		
No		14 (82)
Gestation (weeks)	40.1 (39–41)	
Type of birth		
Spontaneous vaginal		14 (82)
Vacuum assisted vaginal		3 (18)
Epidural and oxytocin		
Yes		12 (71)
Reported edema		
Prenatal		
Yes		12 (71)
Postpartum		
Yes		12 (71)
Newborn sex		
Female		11 (65)
Newborn birth weight (grams)	3442 (2950–3940)	
Timing of first feed		
In first hour		16 (94)

*includes married, living together, common law.

### Intravenous fluids

Intravenous fluids were administered at a set rate via a pump at one hospital and thus mothers (n = 11) seemed to receive IV fluids according to an established standard of care. At the second hospital (n = 2) pumps were not used. Of the final sample, 13 mothers had IV fluids and 4 mothers did not. Table 3 shows the amounts of fluids administered.

**Table 3:** **Maternal IV fluids administered**

**Particulars**	**Mean**	**+/−SD(range)**	n*
Prenatal amount (mls)	1883	+/−872(550–3300)	13
Postpartum amount (mls)	904	+/−523 (250–2150)	13
Total IV fluids received (mls)	2787	+/−1044 (1050–4400)	13

*Participants with no IV fluids = 4.

### Breast and areola edema

**Figure 1:**
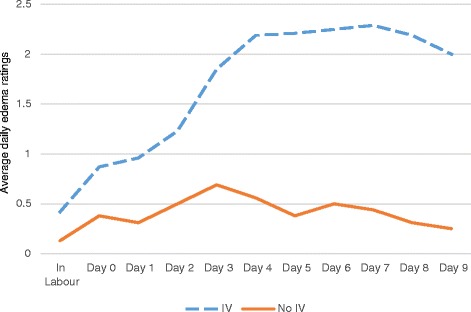
Line graph showing average level of breast edema in participants with Intravenous (IV) fluids vs. participants without IV fluids. Participants with IV fluids n = 13, participants with no IV fluids n = 4. Edema was measured on a daily basis in each breast and areolar of participants. An average daily score for each participant was then calculated and a line graph developed. This graph shows higher average daily edema ratings in participants who received IV fluids as compared to participants who did not receive IV fluids. The blue dashed line represents participants who received IV fluids while the orange solid line represents participants who did not receive IV fluids.

Breast edema was the most compelling variable of the data collected, and seemed to highlight the differences in postpartum breast swelling between participants who had IV fluids and those who did not have IV fluids. The primary researcher, SK-M felt a noticeable difference in breast firmness between participants who had IV fluids and those who had not. When no IV fluids had been given to participants, their breasts felt noticeably softer than breasts of participants who had IV fluids. Participants who had no IV fluids had little to no edema, whereas participants with IV fluids would often have very deep pitting edema. Administration of any amount of IV fluids appeared to make postpartum breast edema worse (see Figure 1). Palpation proved to be a more accurate way of assessing postpartum breast edema than visualization.

Participants who had IV fluids had edema scores that paralleled their maternal breast self-assessment scores, suggesting that these participants may be more aware of the fullness associated with edema than that associated with lactogenesis II. Participants who did not have IV fluids had a spike in the maternal breast self-assessment scores on day 3, which would coincide with the timing of lactogenesis II, and these scores did not follow the pattern set by their breast edema scores. Of note, 38 to 53 percent of participants with IV fluids had moderate to very deep edema as late as Day 8 and Day 9 postpartum which suggests that postpartum breast edema does not self-resolve within a day or two of birth. Of the participants who received no IV fluids, none had moderate to very deep edema. See Additional file 1.

### Maternal breast self-assessment

**Figure 2:**
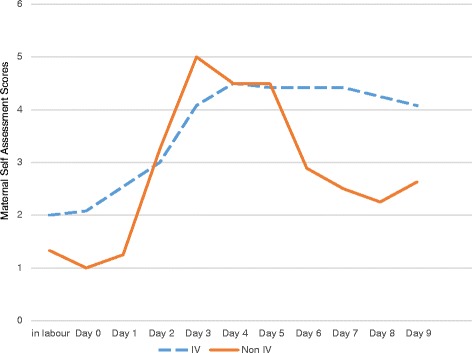
Line graph showing average maternal self-assessment scores (MSA) in participants with Intravenous (IV) fluids vs. participants without IV fluids. Participants with IV fluids n = 13, participants with no IV fluids n = 4. Participants were asked to self-assess breast fullness and tenderness for each breast on a daily basis. An average daily score for each participant was then calculated and a line graph developed. This graph shows participants who had IV fluids rated their breasts more firm and tender for a longer period of time than participants who did not receive IV fluids. The blue dashed line represents participants who received IV fluids while the orange solid line represents participants who did not receive IV fluids.

Participants who had no IV fluids had breasts that followed an expected pattern of fullness, peaking around postpartum Day 3 and 4, and then starting to subside indicating they were feeling and rating engorgement. Participants who had IV fluids had longer periods of extreme fullness, and generally rated their breasts as fuller than their non IV co-participants at each time point, which suggests they may have been feeling and rating edema. It is important to note that 67–69% of participants who received IV fluids experienced firm and tender or very firm and very tender breasts on postpartum Days 7, 8 and 9, (when, according to the literature one would expect engorgement to be resolving [9]) as compared to 0% of participants who did not receive IV fluid. See Additional file 2 and Figure 2.

### Lactogenesis II

This variable was assessed with self-reporting by participants who were advised that signs of lactogenesis II were fuller, heavier breasts, tender breasts, and breasts that may leak milk [20]. By Day 2 (from 48–72 hours following birth), 53% of participants reported that lactogenesis II was starting. By Day 3 (from 72–96 hours following birth), 65% of participants reported that lactogenesis II had occurred. By postpartum Day 6 (from 144–168 hours following birth), all participants reported that lactogenesis II had occurred, however some of the participants found their milk supply was not adequate due to babies not settling after feeds, not having adequate output, test weights done at a breastfeeding clinic indicating inadequate milk transfer, and newborn weight loss, and they needed to supplement their babies and enhance milk production with extra stimulation to their breasts (hand expressing/pumping).

### Newborn weight measurement

Newborns were weighed on a daily basis on an Ultrascale MBSC-55 Digital Scale which was standardized prior to every weight measured. This variable was included to help understand the possible cause of postpartum breast swelling. The rationale was if the newborn was not gaining weight then maternal breast swelling was more likely due to edema rather than engorgement. If the newborn was gaining weight, participants may be more likely to be experiencing lactogenesis II and engorgement. Because of inclusion criteria we were not expecting problems with weight gain. By Day 3 postpartum most newborns (n = 10) started gaining weight and by postpartum Day 4 all newborns measured (2 had missing data) had either stabilized weight loss (n = 2) or started gaining weight (n = 13).

### Pumping and supplementing

Changes in postpartum breast swelling did not appear to be affected by pumping and supplementing, but this may have been due to the small sample size. In a larger study observing pumping and supplementing may lead to a better understanding of factors affecting postpartum breast swelling. Consideration of the mediating and moderating effects of pumping and supplementing on postpartum breast swelling should be taken into account.

### Peripheral limb edema

Due to the late inclusion of this measurement, it was only measured on the last 2 participants and therefore insufficient data were available to draw any conclusions. This variable would provide useful information in a larger study by providing further information on the timing and resolution of peripheral edema as compared to breast edema.

### Variables that proved to be impractical

Several variables were measured that were determined to be either insignificant or not practical to use. There were different reasons for eliminating these measurements, in some cases the measurement did not give us information that seemed valuable, while in other cases the measurement did not give us measurements that were readable. These variables included milk maturation levels (Milk Maturation Index of Colostrum and Milk – MICAM), nipple diameter and height with rulers, and latching by self-reporting from mothers. The test to measure milk maturation (MICAM) proved to be difficult to read and interpret, and it did not appear to relate to amount of milk available according to the primary researcher, SK-M. In all cases milk appeared to mature as expected, however some of these babies required supplementation as mentioned above, leading to the question of adequacy of milk volume or babies not being able to transfer milk as needed. Our main concern with MICAM however was its difficulty to read and interpret. Nipple diameter and shape did vary over time; however, these measurements did not appear to change in a meaningful way. Self-reports of latching were not meaningful due to the many factors that influence why babies latch (or not), some of which seemed unrelated to breast swelling (e.g., going back to breast for a baby who had been supplemented with a bottle). In a larger study about breast swelling we would not recommend the use of these variables.

### Linear regression – relationship between IV fluids received and breast edema

Due to the small sample size in this pilot study, we were unable to use inferential statistics to determine whether there is an association between the amount and timing of IV fluids given during labour and postpartum breast swelling. On discussion with a statistician from the University of Ottawa, it was decided that a linear regression model could be run with the average daily edema scores for each participant (repeated measures) and any IV fluids as a determining factor for edema. All 17 data sets were used for this analysis, 17 participants, with 11 data capture points, and 8 cases of missing data (17 × 11 = 187 – 8 = 179). The linear regression model showed a moderate effect of statistical significance between participants who received IV fluids (*M* = .75, *SD* = .43, *n* = 179) and average edema per day (*M =* 1.35*, SD =* 1.23*, n =* 179) with *r* = .437 and p < .001.). For a larger study the regression analysis should include other covariates which may contribute to edema.

## Discussion

The results of this pilot study suggest that a larger observational cohort study about the influence of IV fluids given in the perinatal period on postpartum breast swelling would be feasible and worthwhile. The preliminary results suggest that mothers who received IV fluids during and after labour experienced increased postpartum breast swelling and the increased swelling contributed to breast pain. It was unclear whether the amount of IV fluid would impact edema levels and this should be tracked in a larger study. However, to adequately develop strategies to address postpartum breast swelling, more in-depth research is required.

**Figure 3:**
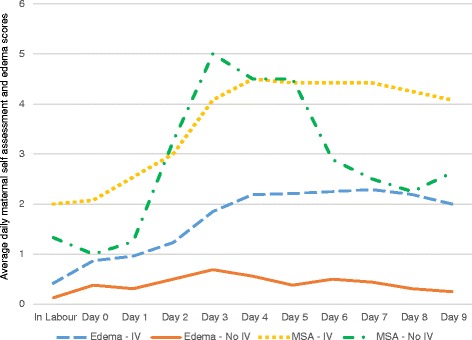
Line graph showing average maternal breast self-assessment (MSA) scores and average edema ratings. Participants with Intravenous (IV) fluids, n = 13, participants with no IV fluids n = 4. This graph contains the data from both Figures 1 and 2. The graph shows the changes in both average daily breast and areola edema scores as well as average daily maternal self-assessment scores. The light blue dashed line shows average daily edema levels for participants who received intravenous (IV) fluids, the solid orange line shows average daily edema levels for participants who did not receive IV fluids. The yellow dotted line shows average daily maternal self-assessment scores for participants who received IV fluids and the green dash-dot line shows average daily maternal self-assessment scores for participants who did not receive IV fluids.

Participants in this pilot study who received IV fluids appeared to be less aware of the fullness associated with lactogenesis II, even though they stated that their breasts were full and tender. The maternal breast self-assessment scores of participants with IV fluids paralleled their edema scores, while participants who did not have IV fluids had a spike in the maternal breast self-assessment scores on day 3, which would coincide with the timing of lactogenesis II. Their scores did not follow the pattern set by their breast edema scores (see Figure 3).

Clinicians need evidence to understand postpartum breast swelling and to develop strategies to help breastfeeding women. Mistaking breast edema for lactogenesis II may give mothers and their clinicians incorrect information regarding milk production. If all breast swelling is considered to represent an increase in milk volume without considering the possible contributing effect of edema on breast swelling, inadequate milk supply and/or transfer might be missed. Inappropriate interventions for postpartum breast swelling, e.g. pumping to remove milk when swelling is due to edema may also be implemented, which may result in making breast edema worse.

Strengths of this pilot study included prospective data collection, a determination of variables worth measuring, insight gained by observations, and the consistency of a single data collector. The study also demonstrates that measuring edema by assessing underlying breast tissue with palpation rather than visualizing skin rebound provided a better assessment of edema.

Limitations of this study included the homogeneity of the sample, a high attrition rate, lack of blinding, and a modification to how edema was measured during the study, which may have led to a Type II error. With respect to cases where IV fluids were run without the use of a pump, there may have been a Hawthorne effect [23] with nurses possibly slowing down IV rates due to the study. Another limitation of the study was the absence of peripheral edema measurements. By the end of the study it was recognized that peripheral edema should have been measured to help track overall edema. Peripheral edema should be measured in the same way as breast and areolar edema is measured, using palpation and a rating scale. For a larger study these strengths and limitations should be taken into account. To avoid a potential source of bias the research assistants collecting data in a larger study should be blinded to whether the participants had IV fluids or not. However, if participants are collecting any of the data blinding would not be possible. A larger study should also include participants who are multiparous, have had Caesarian sections or have had an induced labour. Data collection was completed by the same researcher, which led to consistency of data capture, but no protocol was trialed with multiple data collectors. Data collection was labour intensive with one researcher doing all the data collection. Therefore, inter-rater reliability of the measures and the feasibility of use among multiple data collectors were not evaluated.

Prospective data collection was completed for 10 days by the researcher ensuring internal validity. This intense data collection period helped the researcher understand the daily changes to breast tissue and how it influenced maternal comfort and breastfeeding. Prospective data collection is considered more rigorous than retrospective data collection [24]. For a larger study prospective data collection would be recommended even with normally charted variables such as IV fluids.

This pilot study provided the opportunity to test several data collection measures to determine which are useful and the most feasible and which are unnecessary for the purposes of a larger study on the same topic. Some variables provided insight into the research question while others did not. Discovering which variables are worth measuring in a larger study is a significant contribution from this pilot study. The variables to include are IV fluids, breast and areola edema, maternal breast self-assessment scores, daily newborn weights, onset of lactogenesis II, and peripheral edema. Pumping and supplementing may also be useful to track. Other variables that were not included in this pilot study but may be useful are newborn jaundice levels and breastfeeding rates (i.e., duration and exclusivity) to determine if IV fluids are related to jaundice levels and breastfeeding outcomes.

Clinical practice has changed rapidly over the years, however, research evidence is not available to support change in some areas and ensure that negative outcomes from newer practices are minimized. Given the increased use of epidurals and IV fluids [25], further research is needed to increase knowledge about how maternal IV fluid administration can influence postpartum breast swelling. Recent studies have highlighted some negative outcomes mothers and babies experience in the early postpartum period with regards to a delay in lactogenesis II and initial weight loss due to IV fluids [16,26]. If the results from these studies and this pilot study can be reproduced and validated in larger studies, it could provide strong evidence to guide clinical practice and increase health care providers’ understanding of the effects of IV fluids.

While the administration of IV fluids may be necessary [27,28], nursing care should include instruction on the potential effects of IV fluids on breastfeeding and what mothers can do to minimize the postpartum effects of IV fluids. Further research is required to determine which interventions (e.g. cold packs, cabbage leaf applications, heat, massage, and reverse pressure softening) would be most useful when swelling is due to IV fluids.

Given the potential detrimental effects of postpartum breast swelling, health care professionals working with women in labour should be aware of the apparent adverse effects of IV fluids, e.g. falsely elevating birth weights, delaying lactogenesis II [16], and possibly contributing to postpartum breast swelling. IV fluid administration should be closely monitored whether pumps are used or not. It is expected that a larger study would provide guidance for policy and procedures relating to the safe use of IV fluids.

Postpartum health care providers should be aware that IV fluids given to women during labour may have an influence on breast swelling in the first few weeks postpartum. Anticipatory guidance should be given to women prior to discharge from hospital, as the timing of breast swelling suggests that it would occur after hospital discharge.

A typical general statement regarding engorgement is provided in the "Breastfeeding Matters" [29] booklet which is given to women in Ontario after giving birth. This advice appears to be simplistic, and women may require more detailed information to adequately deal with postpartum breast swelling in the postpartum period. For example, Breastfeeding Matters states that swelling (engorgement) lasting for 24 to 48 hours is normal, and it suggests that prevention of engorgement by breastfeeding at least 8 times per day, feeding at both breasts with each feeding, and breast massage is the best approach [29]. Women need to be made aware that not all postpartum breast swelling is associated with breast milk production [3] and frequent feedings may not be enough to relieve swelling.

## Conclusion

Breastfeeding is physiologically normal behaviour, yet women encounter challenges associated with breastfeeding that may lead them to wean prematurely. One of the main reasons mothers give for weaning is breast and nipple pain associated with breastfeeding. Postpartum breast swelling can aggravate this pain. As maternity practices change and women commonly receive IV fluids prior to regional analgesia [27], research is needed to increase awareness and understanding about the undesirable side effects of IV fluid administration.

To support breastfeeding women, clinicians need evidence about the effects of maternal IV fluids in the postpartum period. This pilot study was completed to assess whether there might be a relationship between breast swelling and maternal IV fluids, to determine if results indicate that further research is warranted, and to determine which variables would be useful to track in a larger study. In the end, the findings demonstrated that mothers in this pilot study who received IV fluids in labour and postpartum had higher levels of breast edema. These results merit further investigation in a larger prospective research study.

## Additional files

Additional file 1:
**Breast edema as measured in each breast at 11 time points.**
Additional file 2:
**Maternal breast self-assessment as reported in each breast by participants.**

## References

[1] Health Canada. Breastfeeding Initiation in Canada: Key Stastics and Graphics (2009–2010). http://www.hc-sc.gc.ca/fn-an/surveill/nutrition/commun/prenatal/initiation-eng.php. Accessed 14 March 2014

[2] Statistics Canada. Breastfeeding, 2009. 2011 http://www.statcan.gc.ca/pub/82-625-x/2010002/article/11269-eng.htm. Accessed 14 March 2014

[3] Lawrence RA, Lawrence RM (2011). Breastfeeding: a guide for the medical profession.

[4] Mangesi L, Dowswell T (2010). Treatments for breast engorgement during lactation. Cochrane Database Syst Rev.

[5] Newton M, Newton NR (1951). Postpartum engorgement of the breast. Am J Obstet Gynecol..

[6] Cirolia B (1996). Understanding edema. Nursing..

[7] Cotterman KJ (2004). Reverse pressure softening: a simple tool to prepare areola for easier latching during engorgement. J Hum Lact..

[8] Miller V, Riordan J (2004). Treating postpartum breast edema with areolar compression. J Hum Lact..

[9] Hill P, Humenick S (1994). The occurrence of breast engorgement. J Hum Lact..

[10] Grant MJ, Booth A (2009). A typology of reviews: an analysis of 14 review types and associated methodologies. Health Info Libr J..

[11] Moon J, Humenick S (1989). Breast engorgement: contributing variables and variables amenable to nursing intervention. J Obstet Gynecol Neonatal Nurs..

[12] Academy of Breastfeeding Medicine Protocol Committee, Berens P. ABM clinical protocol #20: Engorgement. Breastfeeding Medicine: Breastfeed Med. 2009;4:111–113. 10.1089/bfm.2009.9997 19517578

[13] Gonik B, Cotton D, Spillman T, Abouleish E, Zavisca F (1985). Peripartum colloid osmotic pressure changes: effects of controlled fluid management. Am J Obstet Gynecol..

[14] Cunningham F, Leveno K, Bloom S, Hauth J, Gilstrap L, Wentstrom K (2005). Williams Obstetrics.

[15] Lewis S, Heitkemper M, Dirksen S, Barry MA, Goldsworthy S, Goodridge D (2012). Medical-surgical nursing in Canada: Assessment and management of clinical problems.

[16] Noel-Weiss J, Woodend AK, Peterson WE, Gibb W, Groll DL (2011). An observational study of associations among maternal fluids during parturition, neonatal output, and breastfed newborn weight loss. Int Breastfeed J..

[17] Norman G, Streiner D (1998). PDQ epidemiology.

[18] O'Sullivan SB, Schmitz TJ (2007). Physical rehabilitation: Assessment and treatment.

[19] Brodovicz KG, McNaughton K, Uemura N, Meininger G, Girman CJ, Yale SH (2009). Reliability and feasibility of methods to quantitatively assess peripheral edema. Clinical Medicine & Research.

[20] Lauwers J, Swisher A (2005). Counseling the Nursing Mother: A Lactation Consultant's Guide.

[21] Chapman DJ, Perez-Escamilla R (2000). Maternal perception of the onset of lactation is a valid, public health indicator of lactogenesis stage II. J Nutr.

[22] Humenick S (1987). The clinical significance of breastmilk maturation rates. Birth.

[23] Schwartz D, Fischhoff B, Krishnamurti T, Sowell F (2013). The Hawthorne effect and energy awareness. Proc Natl Acad Sci U S A.

[24] Nagurney JT, Brown DF, Sane S, Weiner JB, Wang AC, Chang Y (2005). The accuracy and completeness of data collected by prospective and retrospective methods. Acad Emerg Med..

[25] Better Outcomes Registry and Network Ontario. Perinatal health presentation 2011–2012. 2013. http://www.bornontario.ca/en/resources/reports/lhin-regional-reports/. Accessed 10 Apr 2015.

[26] Chantry CJ, Nommsen-Rivers LA, Peerson JM, Cohen RJ, Dewey KG (2011). Excess weight loss in first-born breastfed newborns relates to maternal intrapartum fluid balance. Pediatrics.

[27] Hofmeyr JG, Cyna AM, Middleton P (2010). Prophylactic intravenous preloading for regional analgesia in labour. Cochrane Database of Syst Rev.

[28] Cyna AM, Andrew M, Emmett RS, Middleton P, Simmons SW (2006). Techniques for preventing hypotension during spinal anaesthesia for caesarean section. Cochrane Database Syst Rev.

[29] Best Start. Breastfeeding Matters, an important guide to breastfeeding for women and their families. 2013. http://www.beststart.org/resources/breastfeeding/breastfeeding_matters_EN_LR.pdf. Accessed 10 April 2015.

