# Supplementary material for: Maternal intravenous fluids and postpartum breast changes: a pilot observational study
Source: Int Breastfeed J. 2015 Jun 2;10:18. doi: 10.1186/s13006-015-0043-8 (PMC4480510; doi:10.1186/s13006-015-0043-8)
Supplement: Additional file 1: — Breast edema as measured in each breast at 11 time points. [file 13006_2015_43_MOESM1_ESM.docx]

**Additional file 1**

**Breast Edema as Measured in Each Breast at 11 Time Points. (N = 17)**

| Frequencies (in number experienced) | | | | | | | | | | | |
| --- | --- | --- | --- | --- | --- | --- | --- | --- | --- | --- | --- |
|  | | No edema (0) | | Slight edema (1+) | | Moderate edema (2+) | | Deep edema (3+) | | Very deep edema (4+) | |
| Timing | | R | L | R | L | R | L | R | L | R | L |
| In labour | IV | 7 | 7 | 6 | 6 | 0 | 0 | 0 | 0 | 0 | 0 |
|  | No IV | 3 | 4 | 1 | 0 | 0 | 0 | 0 | 0 | 0 | 0 |
| Day 0  (Birth) | IV | 5 | 4 | 4 | 5 | 4 | 4 | 0 | 0 | 0 | 0 |
|  | No IV | 3 | 3 | 1 | 1 | 0 | 0 | 0 | 0 | 0 | 0 |
| Day 1 | IV | 7 | 5 | 2 | 6 | 4 | 1 | 0 | 1 | 0 | 0 |
|  | No IV | 3 | 3 | 1 | 1 | 0 | 0 | 0 | 0 | 0 | 0 |
| Day 2 | IV | 2 | 3 | 8 | 5 | 0 | 2 | 3 | 3 | 0 | 0 |
|  | No IV | 3 | 1 | 1 | 3 | 0 | 0 | 0 | 0 | 0 | 0 |
| Day 3* | IV | 2 | 2 | 3 | 3 | 4 | 4 | 0 | 1 | 3 | 2 |
|  | No IV | 2 | 1 | 1 | 3 | 1 | 0 | 0 | 0 | 0 | 0 |
| Day 4* | IV | 0 | 0 | 4 | 4 | 3 | 2 | 0 | 1 | 5 | 5 |
|  | No IV | 1 | 3 | 3 | 1 | 0 | 0 | 0 | 0 | 0 | 0 |
| Day 5* | IV | 0 | 0 | 3 | 3 | 4 | 2 | 0 | 4 | 5 | 3 |
|  | No IV | 2 | 3 | 2 | 1 | 0 | 0 | 0 | 0 | 0 | 0 |
| Day 6* | IV | 2 | 1 | 2 | 1 | 2 | 4 | 1 | 2 | 5 | 4 |
|  | No IV | 2 | 3 | 1 | 1 | 1 | 0 | 0 | 0 | 0 | 0 |
| Day 7* | IV | 1 | 0 | 2 | 3 | 3 | 3 | 2 | 2 | 4 | 4 |
|  | No IV | 2 | 2 | 2 | 2 | 0 | 0 | 0 | 0 | 0 | 0 |
| Day 8** | IV | 2 | 1 | 1 | 2 | 3 | 4 | 2 | 2 | 3 | 3 |
|  | No IV | 2 | 3 | 2 | 1 | 0 | 0 | 0 | 0 | 0 | 0 |
| Day 9 | IV | 2 | 1 | 2 | 2 | 4 | 2 | 2 | 5 | 2 | 2 |
|  | No IV | 2 | 3 | 1 | 1 | 1 | 0 | 0 | 0 | 0 | 0 |

*Participants with IV fluids = 13, Participants with no IV fluids = 4*

*Note that participants could experience different levels of edema in their breasts on the same day* *n = 16 **n = 15 for right breast, n = 16 for left breast
