# Supplementary material for: Maternal intravenous fluids and postpartum breast changes: a pilot observational study
Source: Int Breastfeed J. 2015 Jun 2;10:18. doi: 10.1186/s13006-015-0043-8 (PMC4480510; doi:10.1186/s13006-015-0043-8)
Supplement: Additional file 2: — Maternal breast self-assessment as reported in each breast by participants. [file 13006_2015_43_MOESM2_ESM.docx]

**Additional file 2**

**Maternal Breast Self-Assessment as Reported in Each Breast by Participants. (N = 17)**

| Frequencies (in number experienced) | | | | | | | | | | | | | |
| --- | --- | --- | --- | --- | --- | --- | --- | --- | --- | --- | --- | --- | --- |
|  | | Soft – no change | | Slight Change | | Firm, non-tender | | Firm,  beginning tenderness | | Firm, tender | | Very firm, very tender | |
| Timing | | R | L | R | L | R | L | R | L | R | L | R | L |
| In Labour* | IV | 4 | 4 | 4 | 4 | 5 | 5 | 0 | 0 | 0 | 0 | 0 | 0 |
|  | No IV | 2 | 2 | 1 | 1 | 0 | 0 | 0 | 0 | 0 | 0 | 0 | 0 |
| Day 0  (Birth) | IV | 3 | 3 | 4 | 4 | 6 | 6 | 0 | 0 | 0 | 0 | 0 | 0 |
|  | No IV | 4 | 4 | 0 | 0 | 0 | 0 | 0 | 0 | 0 | 0 | 0 | 0 |
| Day 1 | IV | 1 | 1 | 6 | 6 | 5 | 5 | 1 | 1 | 0 | 0 | 0 | 0 |
|  | No IV | 3 | 3 | 1 | 1 | 0 | 0 | 0 | 0 | 0 | 0 | 0 | 0 |
| Day 2 | IV | 1 | 1 | 2 | 2 | 3 | 4 | 4 | 3 | 2 | 2 | 0 | 0 |
|  | No IV | 1 | 1 | 0 | 0 | 0 | 1 | 3 | 2 | 0 | 0 | 0 | 0 |
| Day 3 | IV | 0 | 0 | 0 | 0 | 1 | 1 | 5 | 5 | 6 | 6 | 0 | 0 |
|  | No IV | 0 | 0 | 0 | 0 | 0 | 0 | 1 | 1 | 3 | 2 | 0 | 1 |
| Day 4 | IV | 0 | 0 | 0 | 0 | 0 | 0 | 3 | 3 | 7 | 7 | 2 | 2 |
|  | No IV | 1 | 0 | 0 | 1 | 0 | 0 | 0 | 0 | 1 | 1 | 2 | 2 |
| Day 5 | IV | 0 | 0 | 1 | 1 | 0 | 0 | 2 | 2 | 6 | 6 | 3 | 3 |
|  | No IV | 0 | 0 | 0 | 0 | 0 | 1 | 1 | 1 | 3 | 2 | 0 | 0 |
| Day 6 | IV | 0 | 0 | 0 | 0 | 1 | 1 | 2 | 2 | 6 | 6 | 3 | 3 |
|  | No IV | 0 | 0 | 2 | 2 | 1 | 1 | 0 | 1 | 1 | 0 | 0 | 0 |
| Day 7 | IV | 0 | 0 | 0 | 0 | 1 | 1 | 2 | 2 | 6 | 6 | 3 | 3 |
|  | No IV | 1 | 1 | 1 | 1 | 1 | 1 | 1 | 0 | 0 | 0 | 0 | 0 |
| Day 8 | IV | 0 | 0 | 1 | 1 | 0 | 0 | 2 | 2 | 7 | 7 | 2 | 2 |
|  | No IV | 1 | 1 | 1 | 1 | 2 | 2 | 0 | 0 | 0 | 0 | 0 | 0 |
| Day 9 | IV | 1 | 0 | 1 | 1 | 1 | 1 | 1 | 2 | 4 | 6 | 4 | 2 |
|  | No IV | 1 | 1 | 1 | 1 | 0 | 1 | 2 | 1 | 0 | 0 | 0 | 0 |

*Participants with IV fluids = 13, Participants with no IV fluids = 4*

*Note participants could experience different levels of fullness in separate breasts * n = 16*
